# High Mobility Group Box-1 (HMGB1) Participates in the Pathogenesis of Alcoholic Liver Disease (ALD)*

**DOI:** 10.1074/jbc.M114.552141

**Published:** 2014-06-13

**Authors:** Xiaodong Ge, Daniel J. Antoine, Yongke Lu, Elena Arriazu, Tung-Ming Leung, Arielle L. Klepper, Andrea D. Branch, Maria Isabel Fiel, Natalia Nieto

**Affiliations:** From the ‡ Division of Liver Diseases, Department of Medicine and; ¶ Department of Pathology, Icahn School of Medicine at Mount Sinai, New York, New York 10029 and; § Medical Research Council Centre for Drug Safety Science, Molecular and Clinical Pharmacology, University of Liverpool, Sherrington Buildings, Ashton Street, Liverpool L69 3GE, United Kingdom

**Keywords:** Alcohol, Hepatocyte, Inflammation, Liver, Post-translational Modification (PTM), High Mobility Group Box 1

## Abstract

Growing clinical and experimental evidence suggests that sterile inflammation contributes to alcoholic liver disease (ALD). High mobility group box-1 (HMGB1) is highly induced during liver injury; however, a link between this alarmin and ALD has not been established. Thus, the aim of this work was to determine whether HMGB1 contributes to the pathogenesis of ALD. Liver biopsies from patients with ALD showed a robust increase in HMGB1 expression and translocation, which correlated with disease stage, compared with healthy explants. Similar findings were observed in chronic ethanol-fed wild-type (WT) mice. Using primary cell culture, we validated the ability of hepatocytes from ethanol-fed mice to secrete a large amount of HMGB1. Secretion was time- and dose-dependent and responsive to prooxidants and antioxidants. Selective ablation of *Hmgb1* in hepatocytes protected mice from alcohol-induced liver injury due to increased carnitine palmitoyltransferase-1, phosphorylated 5′AMP-activated protein kinase-α, and phosphorylated peroxisome proliferator-activated receptor-α expression along with elevated LDL plus VLDL export. Native and post-translationally modified HMGB1 were detected in humans and mice with ALD. In liver and serum from control mice and in serum from healthy volunteers, the lysine residues within the peptides containing nuclear localization signals (NLSs) 1 and 2 were non-acetylated, and all cysteine residues were reduced. However, in livers from ethanol-fed mice, in addition to all thiol/non-acetylated isoforms of HMGB1, we observed acetylated NLS1 and NLS2, a unique phosphorylation site in serine 35, and an increase in oxidation of HMGB1 to the disulfide isoform. In serum from ethanol-fed mice and from patients with ALD, there was disulfide-bonded hyperacetylated HMGB1, disulfide-bonded non-acetylated HMGB1, and HMGB1 phosphorylated in serine 35. Hepatocytes appeared to be a major source of these HMGB1 isoforms. Thus, hepatocyte HMGB1 participates in the pathogenesis of ALD and undergoes post-translational modifications (PTMs) that could condition its toxic effects.

## Teaser

**Background:** HMGB1 is a proinflammatory cytokine produced in response to tissue injury, but its role in ALD is unknown.

**Results:** HMGB1 increases; translocates; and undergoes acetylation, phosphorylation, and oxidation in ALD. HMGB1 ablation in hepatocytes protects against steatosis and injury in ALD.

**Conclusion:** HMGB1 plays a key role in ALD.

**Significance:** Dissecting how the increase in HMGB1 causes hepatotoxicity is key for understanding the pathogenesis of ALD.

## Introduction

Alcoholic liver disease (ALD)^4^ is a major cause of morbidity and mortality worldwide. The spectrum of disease ranges from simple fatty liver to steatohepatitis, progressive fibrosis, cirrhosis, and hepatocellular carcinoma. In developed countries, ALD is a major cause of end stage disease that requires liver transplantation. Therefore, identifying proinflammatory proteins that are critical in the pathogenesis of ALD and that could be used as biomarkers of disease progression is vital for diagnostic purposes, for patient stratification, and to improve the quality of life in many patients.

High mobility group box-1 (HMGB1) is a highly conserved eukaryotic non-histone chromosomal protein of 215 amino acids with a tripartite structure composed of two DNA-binding domains (box A and box B) and an acidic C-terminal domain containing aspartate and glutamate residues (1). Upon stimulation, HMGB1 undergoes translocation from the nucleus to the cytoplasm, and it is then secreted via the lysosomal pathway in most cells (2, 3), by exosomes in enterocytes (4, 5), or by the inflammasome in immune cells (6–10). HMGB1 binds the receptor for advanced glycation end products, toll-like receptors-2/4/9, Mac-1, syndecan-1, phosphacan protein-tyrosine phosphatase-ζ/β, and CD24 (11–15).

Initially, it was believed that this non-sequence-specific DNA-binding protein with two nuclear localization signals (NLSs) acted primarily as an architectural element. Among its well known roles in the nucleus, HMGB1 activates or inhibits transcription (16–18); modulates DNA repair (19); promotes nucleoprotein complex assembly (20); facilitates nucleosome disruption and remodeling (21); acts as a cofactor in mammalian base excision repair (22); and participates in recombination (23), suppression of genotoxic stress signaling (24), and maintenance of chromatin in a fluidic state (25).

However, work over the past decade has demonstrated that HMGB1 also has critical effects in the extracellular environment. When passively released from injured or necrotic cells due to loss of membrane integrity (26) or when secreted by activated monocytes and macrophages as a delayed response to lipopolysaccharide (LPS), IL-1, or TNFα, HMGB1 can trigger an inflammatory response (27). Indeed, administration of neutralizing HMGB1 antibodies prevents inflammation and attenuates disease severity (27–29). Thus, HMGB1 is now considered a member of the family of damage-associated molecular patterns that communicate injury, inflammation, and death to neighboring cells.

In all cells, HMGB1 shuttles bidirectionally between the nucleus and cytoplasm via passive and active mechanisms. Under physiological conditions, nuclear import is more favorable than export. However, increased nuclear export in response to stimuli and post-translational modifications (PTMs) drives HMGB1 toward cytoplasmic localization and secretion (1, 30–35). It has been reported that HMGB1 can undergo phosphorylation, acetylation, oxidation, methylation, ADP-ribosylation, and glycosylation (36).

To date, a comprehensive qualitative and quantitative analysis of HMGB1 and its PTMs in ALD is lacking. Because inflammation is a key component contributing to the pathogenesis of ALD and HMGB1 is an early proinflammatory cytokine up-regulated in response to sterile and non-sterile tissue injury, the aims of this study were to determine whether HMGB1 increases in ALD, quantify the extent of HMGB1 translocation from the nucleus to the cytoplasm in ALD, dissect whether HMGB1 induction in hepatocytes is essential for alcohol-induced liver injury, dissect the mechanism involved, identify the PTMs of HMGB1 in ALD, and recognize whether native HMGB1 and post-translationally modified HMGB1 are secreted into the extracellular space under alcohol consumption. To this aim, we used samples from patients with ALD and mouse models of alcohol-induced liver injury along with primary cell culture as well as conditional ablation of *Hmgb1* in hepatocytes *in vivo*.

## EXPERIMENTAL PROCEDURES

### 

#### 

##### General Methodology

Alanine aminotransferase activity and triglycerides were analyzed using kits (Pointe Scientific, Canton, MI). Hematoxylin and eosin (H&E) staining and Sirius red/fast green staining for collagenous proteins and Western blot analysis for HMGB1, nitric-oxide synthase-2, 3-nitrotyrosine, and calnexin were performed as in earlier work (37). All the antibodies used were from Santa Cruz Biotechnology (Santa Cruz, CA). Secreted proteins were concentrated by acetone precipitation. Primary mouse hepatocytes, Kupffer cells, stellate cells, and sinusoidal endothelial cells were isolated as described previously (38). Transmission electron microscopy and measurement of cytochrome P450 2E1 activity, lipid peroxidation, H_2_O_2_, O_2_^⨪^, total and mitochondrial GSH, and TNFα were performed as in previous publications (37, 39). The concentration of HMGB1 was measured by ELISA using a kit from IBL International (Toronto, Canada). LDL and VLDL were measured using a kit from Biovision (Milpitas, CA).

##### Human Samples

Dr. Fiel provided paraffin-embedded archived wedge samples from six de-identified healthy human lobectomy specimens and human liver needle biopsies from 12 patients with clinically proven alcohol-induced liver injury scored according to the Brunt classification (40). Dr. Branch provided serum from 10 de-identified individuals with ALD and from controls. These archived samples were Institutional Review Board-approved. Control sera were also obtained from laboratory personnel. In all cases, no patient information was disclosed.

##### Mice

In the *Hmgb1^fl^*^/^*^fl^* mice, the *Hmgb1^loxP^* allele was created by inserting *loxP* sites within introns 1 and 2 flanking exon 2 of *Hmgb1* (41). These mice were donated by Dr. Billiar (University of Pittsburgh, Pittsburgh, PA) and were bred with *Alb-Cre* mice (The Jackson Laboratory, Bar Harbor, ME) to generate hepatocyte-specific *Hmgb1^fl^*^/^*^fl^Alb-Cre* mice. *Hmgb1^fl^*^/^*^fl^*^−/−^*Alb-Cre*^−^ mice were used as control littermates. *Hmgb1* ablation in hepatocytes was confirmed by genotyping using both tail and liver DNA and by immunohistochemistry (IHC). Minor differences in hepatocyte mRNA expression were found by the donating investigators at baseline between *Hmgb1^fl^*^/^*^fl^Alb-Cre* mice and *Hmgb1^fl^*^/^*^fl^*^−/−^*Alb-Cre*^−^ control littermates (41).

##### Lieber-DeCarli Model of Early Alcohol-induced Liver Injury

To provoke early alcohol-induced liver injury, we used the control and ethanol Lieber-DeCarli diets (42) (Bio-Serv Inc., Frenchtown, NJ), which are equicaloric and have comparable composition of fat (42% of total calories) and protein (16% of total calories). The content of carbohydrates is 42% of total calories (dextrin-maltose) in the control diet and 12% of total calories in the ethanol diet where up to 30% of carbohydrate calories are replaced by ethanol (42). 10-week-old male wild-type (WT) mice or *Hmgb1^fl/fl^Alb-Cre* and *Hmgb1^fl^*^/^*^fl^*^−/−^*Alb-Cre*^−^ control littermates were used in the Lieber-DeCarli model. Mice were acclimatized to the liquid diet regime by feeding them the control diet for 3 days. The percentage of ethanol-derived calories was progressively increased from 10% (1 week) to 20% (1 week), 25% (2 weeks), and 30% (3 weeks). Mice were pair-fed, and body weight was monitored throughout the experiment. Liver and body weights were recorded at the point of sacrifice to calculate the liver-to-body weight ratio for each mouse. Blood was collected by submandibular bleeding under anesthesia, and livers were removed for further analysis. All animals received humane care according to the criteria outlined in the “Guide for the Care and Use of Laboratory Animals” prepared by the National Academy of Sciences and published by the National Institutes of Health.

##### Pathology

The entire mouse left liver lobe was resected, fixed in 10% neutral buffered formalin, and processed into paraffin sections for H&E staining or IHC. Blind analysis by our liver pathologist was used to determine the activity scores according to the Brunt classification (40). Ten 100× fields per liver section from the same mouse were examined for steatosis and necroinflammatory activity, which was scored as follows: 0 for none, 1 for <2 foci per 100× field, 2 for 2–4 foci per 100× field, 3 for 5–10 foci per 100× field, and 4 for >10 foci per 100× field. The density of the necroinflammatory activity was also calculated per mm^2^ per 100× fields.

##### Immunohistochemistry

Archived wedge samples from human lobectomy healthy specimens, human liver needle biopsies from patients with ALD, and control or ethanol-fed mice were immunostained for HMGB1 following standard procedures. The HMGB1 antibody (ab18256) was from Abcam (Cambridge, MA). Immunochemical reactions were developed using the Histostain Plus detection system (Invitrogen). The intensity of the immunostaining was quantified by computer-assisted morphometry assessment. The integrated optical density was calculated in 10 random fields per section containing similar size portal tracts or central veins at 100× using Image-Pro Plus 7.0 Software (Media Cybernetics, Bethesda, MD). The intensity of the red area corresponding to HMGB1-positive staining in the scanned fields was averaged and considered the total HMGB1-positive staining. Next, the software settings were programmed to select all nuclei, and the intensity of the stained red area corresponding to nuclear HMGB1-positive staining in the scanned fields was averaged and considered the nuclear HMGB1-positive staining. The nuclear staining was then subtracted from the total staining to determine the cytoplasmic HMGB1-positive staining. The results were averaged and expressed as -fold change of the controls. The HMGB1 expression ratios were calculated by morphometry analysis as above in 20 fields per slide at 200× magnification and are expressed as nuclear *versus* total and cytoplasmic *versus* total HMGB1 expression. Immunofluorescences were performed on primary hepatocytes, Kupffer cells, stellate cells, and sinusoidal endothelial cells using the same HMGB1 antibody as above followed by Alexa Fluor 488-conjugated goat anti-rabbit IgG secondary antibody (Invitrogen) and visualized by confocal microscopy.

##### Analysis of Serum and Liver HMGB1 by Electrospray Ionization-Liquid Chromatography-Mass Spectrometry (ESI-LC-MS)

All chemicals and solvents used were of the highest available grade (Sigma). Samples were precleared with 50 μl of protein G-Sepharose beads for 1 h at 4 °C. HMGB1 present in serum or liver was immunoprecipitated with 5 μg of rabbit anti-HMGB1 (ab18256) for 16 h at 4 °C as described previously (43). For the analysis of HMGB1 PTMs, free thiol groups within HMGB1 were alkylated for 90 min with 10 mm iodoacetamide at 4 °C. Cysteine residues in disulfide bonds were then reduced with 30 mm dithiothreitol at 4 °C for 1 h followed by alkylation of newly exposed thiol groups with 90 mm *N*-ethylmaleimide at 4 °C for 10 min. Samples were subjected to trypsin (Promega, Madison, WI) or Glu-C (New England Biolabs, Ipswich, MA) digestion according to the manufacturers' instructions and desalted using C_18_ ZipTips (Millipore, Billerica, MA). Characterization of whole protein molecular weights, acetylated lysine residues, or redox modifications on cysteine residues within HMGB1 was performed as described previously by whole protein ESI or tandem mass spectrometry (MS/MS) (44–46) using either an AB Sciex QTRAP 5500 or an AB Sciex TripleTOF 5600 (Sciex Inc., Framingham, MA). Peptide analysis was determined using an AB Sciex QTRAP 5500 equipped with a NanoSpray II source by in-line liquid chromatography using a U3000 HPLC system (Dionex, Sunnyvale, CA), connected to a 180-μm × 20-mm nanoAcquity UPLC C_18_ trap column and a 75-μm × 15-cm nanoAcquity UPLC BEH130 C_18_ column (Waters, Milford, MA) via reducing unions. A gradient from 0.05% TFA (v/v) to 50% acetonitrile, 0.08% TFA (v/v) in 40 min was applied at a flow rate of 200 nl/min. The ion spray potential was set to 2,200–3,500 V, the nebulizer gas was set to 19 L/min, and the interface heater was set to 150 °C.

##### Statistical Analysis

Data were analyzed by a two-factor analysis of variance, and results were expressed as means ± S.E. (*n* = 10–12/group for the human samples and the Lieber-DeCarli model).

## RESULTS

### 

#### 

##### Liver Biopsies from Patients with Acute Alcoholic Steatohepatitis (ASH) Superimposed on ALD and Cirrhosis Show Significant and Progressive Increase in HMGB1 Expression and Translocation from the Nucleus to the Cytoplasm Compared with Wedge Samples from Healthy Human Lobectomy Specimens

**FIGURE 1.**
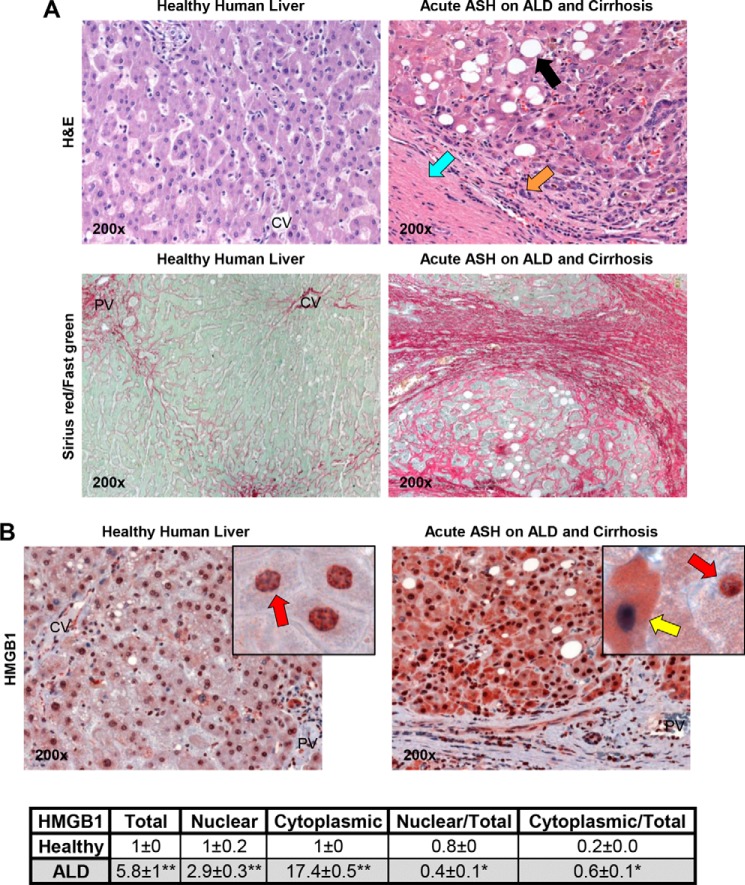
**Liver biopsies from patients with ASH superimposed on ALD and cirrhosis show significant increase in HMGB1 expression and translocation from the nucleus to the cytoplasm compared with wedge samples from healthy human lobectomy specimens.** H&E staining shows significant steatosis (*black arrow*), inflammation (*orange arrow*), and cirrhosis (*blue arrow*), and Sirius red/fast green staining shows total collagen (*red*) in healthy human lobectomy specimens and liver needle biopsies from patients with clinically proven acute ASH superimposed on ALD and cirrhosis (*A*). In HMGB1 IHC, the *red arrows* indicate HMGB1 positive staining in the nucleus and -negative staining in the cytoplasm, and the *yellow arrow* points to HMGB1-negative staining in the nucleus and -positive staining in the cytoplasm (*B*, *top*). Morphometry assessment of total, nuclear, and cytoplasmic HMGB1 expression as well as the ratios of nuclear *versus* total and cytoplasmic *versus* total HMGB1 expression is shown (*B*, *bottom*). *n* = 10/group. *, *p* < 0.05; **, *p* < 0.01 for ALD *versus* healthy.

To determine whether HMGB1 increases in ALD, wedge samples from healthy human lobectomy specimens and liver needle biopsies from patients with clinically proven acute ASH superimposed on ALD and cirrhosis, as shown by H&E and Sirius red/fast green staining (Fig. 1*A*), were analyzed for HMGB1 expression. The IHC analysis demonstrated a robust increase in HMGB1 expression in patients with acute ASH superimposed on ALD and cirrhosis compared with healthy specimens (Fig. 1*B*, *top*). There was a significant increase in HMGB1 expression and in HMGB1 translocation from the nucleus to the cytoplasm that was more apparent in hepatocytes. This was quantified by morphometry assessment and calculation of the increase in total, nuclear, and cytoplasmic HMGB1 along with the ratios of nuclear *versus* total and cytoplasmic *versus* total HMGB1 protein expression (Fig. 1*B*, *bottom*).

**FIGURE 2.**
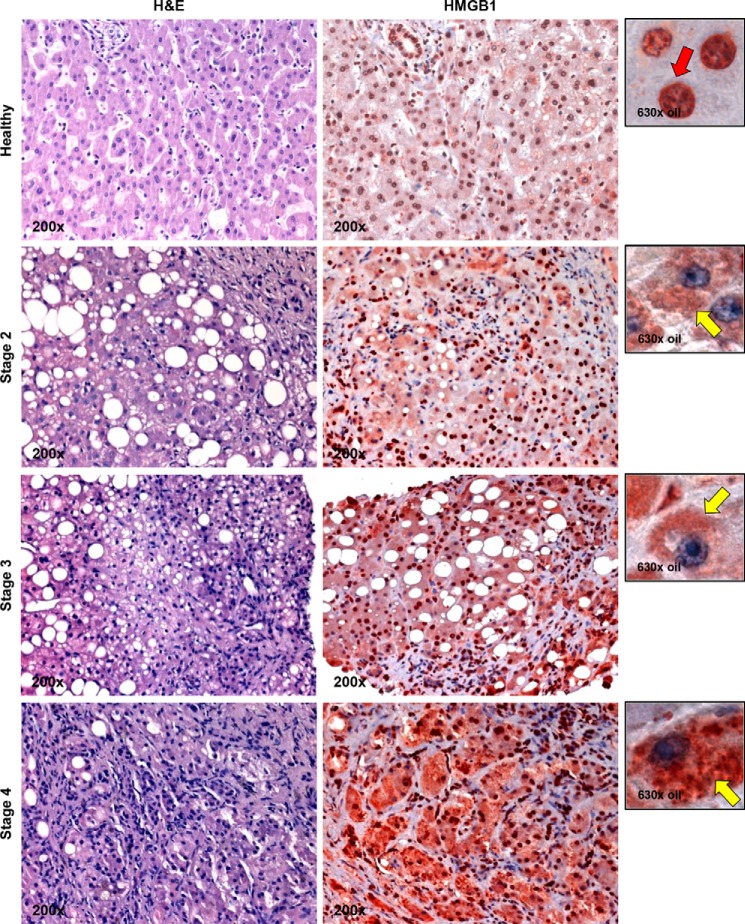
**HMGB1 induction and translocation correlate with disease stage in human ALD.** IHC for HMGB1 in a set of human liver biopsies that are healthy or stage 2 (mild), 3 (significant), and 4 (severe) ALD classified according to the Brunt scoring system (40) is shown. The *red arrow* indicates HMGB1-positive staining in the nucleus and -negative staining in the cytoplasm, and the *yellow arrows* point to HMGB1-negative staining in the nucleus and -positive staining in the cytoplasm.

To dissect whether HMGB1 expression correlated with the extent of alcohol-induced liver damage, we performed IHC analysis for HMGB1 in a set of human liver specimens that ranged from healthy to stages 2 (mild), 3 (significant), and 4 (severe) alcohol-induced liver injury classified according to the Brunt scoring system (40). HMGB1 expression and translocation correlated with the degree of alcohol-induced liver injury (Fig. 2). Overall, these results suggest that patients with ALD show a progressive increase in HMGB1 expression and translocation compared with healthy liver specimens.

##### Alcohol Intake Enhances HMGB1 Expression and Translocation from the Nucleus to the Cytoplasm in WT Mice

**FIGURE 3.**
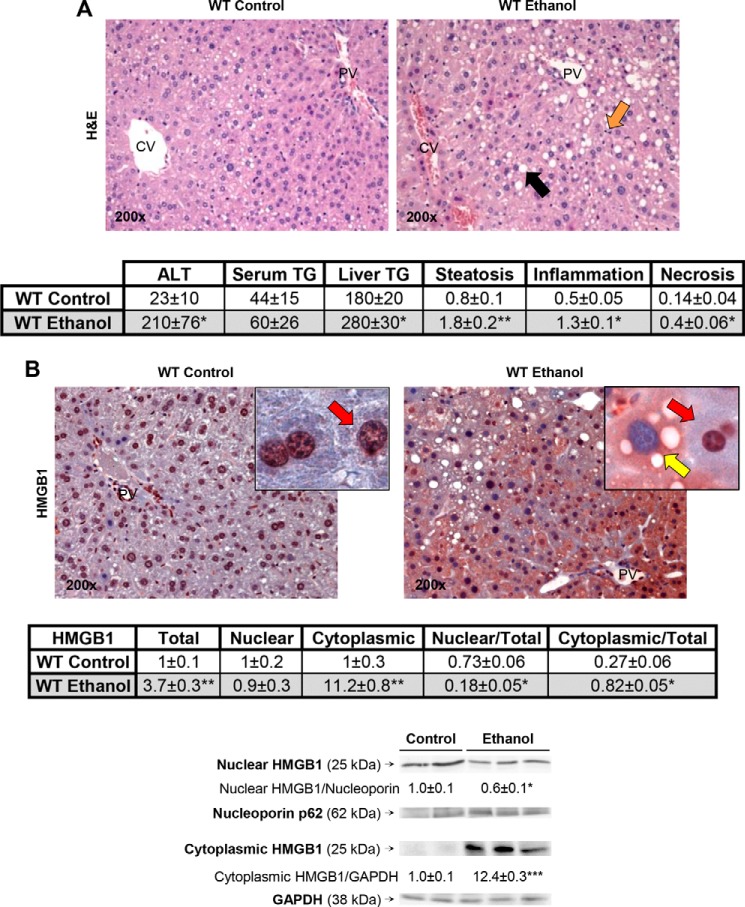
**Alcohol intake enhances HMGB1 expression and translocation from the nucleus to the cytoplasm in WT mice.** WT mice were fed for 7 weeks either the control or the ethanol Lieber-DeCarli diet. H&E staining (steatosis (*back arrow*), inflammation (*orange arrow*)), serum alanine aminotransferase (*ALT*) activity (units/liter), serum triglycerides (*TG*) (mg/dl), and liver triglycerides (mg/g of protein) along with the scores for steatosis, inflammation, and necrosis classified according to the Brunt scoring system (40) are shown (*A*). IHC shows that ethanol feeding increases HMGB1 expression in WT mice. The *red arrows* indicate HMGB1-positive staining in the nucleus and -negative staining in the cytoplasm, and the *yellow arrow* points to HMGB1-negative staining in the nucleus and -positive staining in the cytoplasm (*B*, *top*). Morphometry assessment of total, nuclear, and cytoplasmic HMGB1 as well as the ratios of nuclear *versus* total and cytoplasmic *versus* total HMGB1 expression is shown (*B*, *middle*). Western blot analysis of cytoplasmic and nuclear HMGB1 is also shown (*B*, *bottom*). *n* = 10/group. *, *p* < 0.05; **, *p* < 0.01; ***, *p* < 0.001 for ethanol *versus* control.

To carry out mechanistic studies to understand how ethanol increases HMGB1 expression and nucleocytoplasmic shuttling, mouse models of ALD and genetic approaches are needed. Hence, we questioned whether the changes in HMGB1 expression and cellular localization observed in patients would also occur in mouse models of ALD. To this end, WT mice were fed either the control or the ethanol Lieber-DeCarli diet, a model known to cause early alcohol-induced liver injury (42). Mice fed ethanol showed a 20% increase in the liver-to-body weight ratio compared with mice fed the control diet (not shown). H&E staining revealed more liver injury and steatosis in ethanol-fed mice than in control mice (Fig. 3*A*, *top*). Serum alanine aminotransferase activity and serum and liver triglycerides were higher following ethanol feeding, suggesting increased liver damage compared with mice fed the control diet. Furthermore, the scores for steatosis, inflammation, and necrosis were higher in ethanol-fed mice compared with control mice (Fig. 3*A*, *bottom*). Ethanol induced HMGB1 expression and abundant cytoplasmic localization compared with mice fed control diet (Fig. 3*B*, *top*). This was quantified by morphometry assessment of total, nuclear, and cytoplasmic HMGB1 expression (Fig. 3*B*, *middle*) and by Western blot analysis (Fig. 3*B*, *bottom*), both of which proved a decrease in nuclear *versus* total along with an increase in cytoplasmic *versus* total HMGB1 expression.

To validate that the increase in HMGB1 expression and translocation from the nucleus to the cytoplasm was specific, we used two additional models of alcohol-induced liver injury. For this purpose, we performed the chronic-plus-single binge ethanol feeding model of alcohol-induced liver injury (47). In addition, we obtained samples from a mouse model of alcoholic neutrophilic hepatitis developed in Dr. Tsukamoto's laboratory using a high cholesterol and high fat diet plus intragastric administration of alcohol along with a weekly alcohol binge (49). In both models, IHC analysis showed considerable induction and translocation of HMGB1 in the livers from ethanol-fed mice compared with the respective control mice (not shown). Overall, these results demonstrate that in three mouse models of alcohol-induced liver injury alcohol intake enhances HMGB1 expression and translocation from the nucleus to the cytoplasm in mice similarly to what occurs in humans.

##### Primary Hepatocytes and Kupffer Cells from Control and Ethanol-fed Mice Retain Their Phenotype in Vitro

**FIGURE 4.**
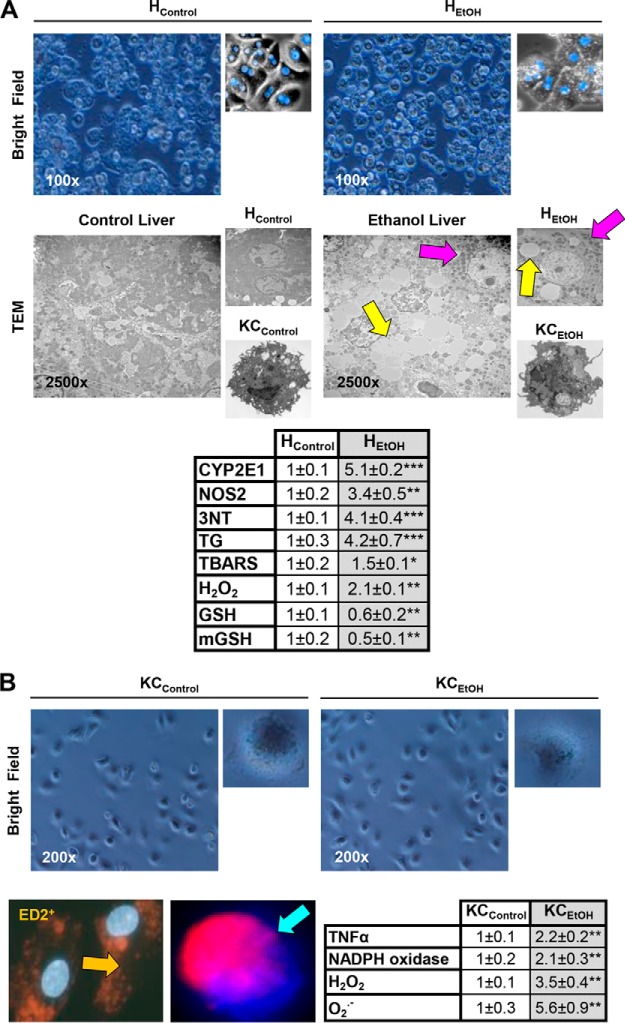

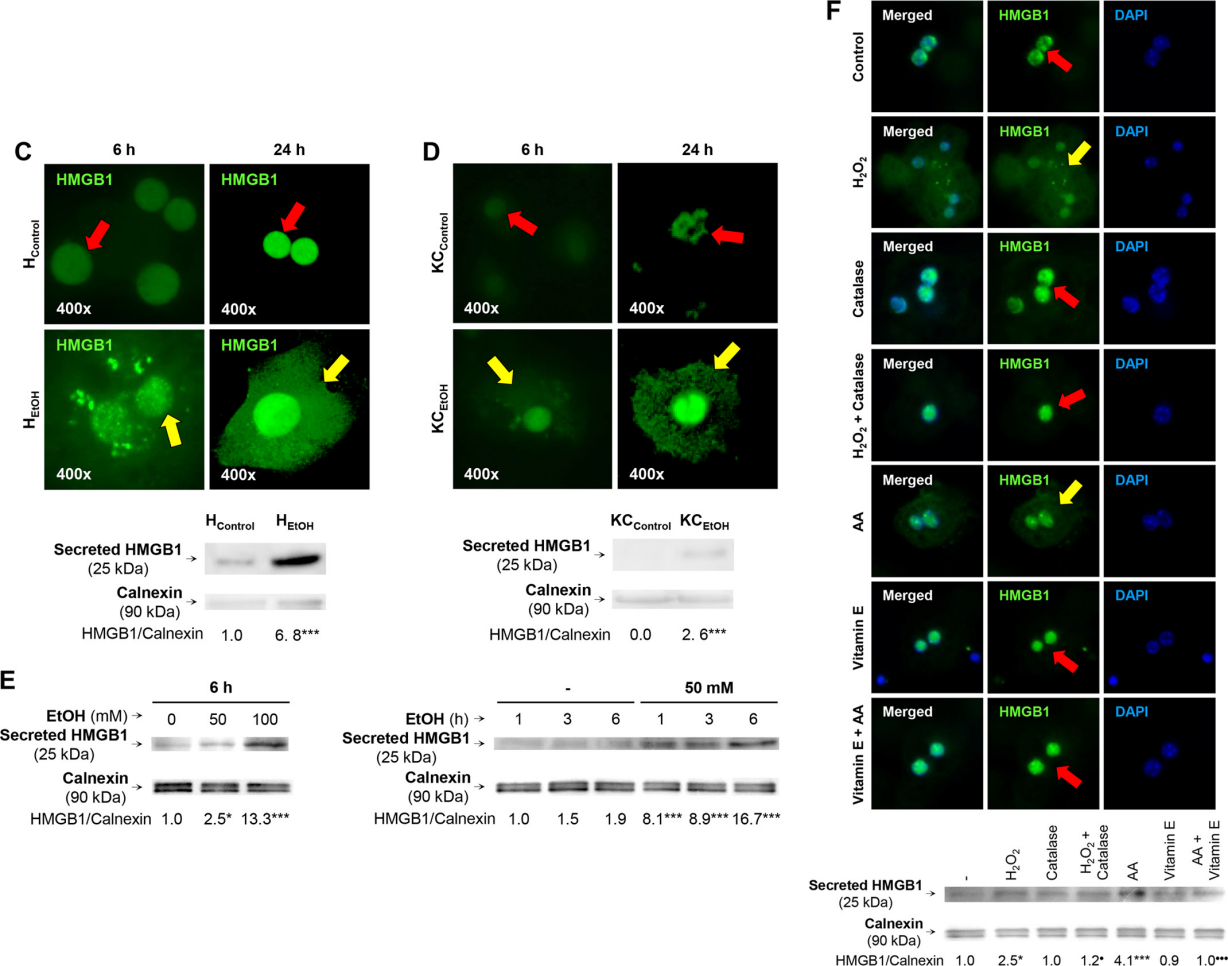
**Primary hepatocytes and Kupffer cells from control and ethanol-fed mice retain their phenotype *in vitro*.** Primary hepatocytes and Kupffer cells were isolated from mice fed the control or the ethanol Lieber-DeCarli diet for 7 weeks (H_Control_, H_EtOH_, KC_Control_, and KC_EtOH_) and cultured for up to 48 h. Bright field pictures along with pictures from hepatocytes fixed and stained with DAPI (*blue*) are shown (*A*, *top*). Transmission electron microscopy pictures from liver, hepatocytes, and Kupffer cells from control and ethanol-fed mice (the *yellow arrows* point to lipid droplets, and the *pink arrows* point to electron-dense mitochondria) are shown (*A*, *middle*). -Fold change of markers of injury in H_Control_ and H_EtOH_ is similar to what occurs *in vivo* (*A*, *bottom*). Bright field pictures from Kupffer cells (*B*, *top*) are shown. Cultured Kupffer cells were fixed and stained with DAPI and ED2 (*orange arrow*) or evaluated for phagocytic activity of fluorescent red nanoparticles (*blue arrow*) (*B*, *bottom left*). -Fold change in markers of injury in KC_Control_ and KC_EtOH_ is similar to what occurs *in vivo* (*B*, *bottom right*). *n* = 6. *, *p* < 0.05; **, *p* < 0.01; ***, *p* < 0.001 for ethanol *versus* control. HMGB1 induction, translocation, and secretion in hepatocytes are critical for alcohol-induced liver injury. Immunofluorescence analysis for HMGB1 expression in H_Control_ and H_EtOH_ (*C*, *top*) and KC_Control_ and KC_EtOH_ (*D*, *top*) at 6 and 24 h post-isolation is shown. The *red arrows* indicate HMGB1-positive staining in the nucleus and -negative staining in the cytoplasm, and the *yellow arrows* point to loss of HMGB1 staining in the nucleus and appearance of a punctate pattern of positive staining in the cytoplasm. Western blot analysis for secreted HMGB1 in equal amounts of protein from the culture medium from H_Control_ and H_EtOH_ (*C*, *bottom*) and KC_Control_ and KC_EtOH_ (*D*, *bottom*) is shown. *n* = 3. ***, *p* < 0.001 for ethanol *versus* control. HMGB1 secretion in hepatocytes is oxidant stress-sensitive. Primary H_Control_ were treated with 0–100 mm ethanol for 6 h or with 50 mm ethanol for 0–6 h. Western blot analysis shows that H_Control_ respond to ethanol treatment in a dose- and time-dependent fashion (*E*). H_Control_ treated with 25 μm H_2_O_2_ or with 30 μm arachidonic acid (*AA*) for 6 h increased HMGB1 expression and secretion as shown by immunofluorescence and Western blot analysis; however, the HMGB1 induction and secretion were prevented by co-treatment with 200 units/ml catalase or with 25 μm vitamin E, respectively (*F*). *n* = 3. *, *p* < 0.05; ***, *p* < 0.001 for treated *versus* control. •, *p* < 0.05; •••, *p* < 0.001 for co-treated *versus* control.

To determine the contribution of each liver cell type to the increase in HMGB1 production, primary hepatocytes and Kupffer cells from control and ethanol-fed mice (H_Control_, H_EtOH_, KC_Control_, and KC_EtOH_) were isolated and cultured up to 48 h. Cells were viable as shown by the typical morphology in the bright field images (Fig. 4, *A* and *B*, *top*) and by the 3-(4,5-dimethylthiazol-2-yl)-2,5-diphenyltetrazolium bromide assay and lactate dehydrogenase leakage (not shown). Transmission electron microscopy also confirmed the ability of H_EtOH_ to retain key features such as the presence of lipid droplets and electron-dense mitochondria and the activated phenotype of KC_EtOH_ (Fig. 4*A*, *middle*). H_EtOH_ showed a significant increase in cytochrome P450 2E1 activity, nitric-oxide synthase-2 expression, 3-nitrotyrosine, triglycerides, lipid peroxidation-end products (measured as thiobarbituric acid-reactive substances) and H_2_O_2_ production, and a decrease in total and mitochondrial GSH when compared with H_Control_ (Fig. 4*A*, *bottom*). Isolated Kupffer cells were identified as ED2^+^ (a marker of Kupffer cells) and showed phagocytosis of fluorescent red nanoparticles. Moreover, KC_EtOH_ displayed higher TNFα expression and NADPH oxidase activity along with O_2_^⨪^ and H_2_O_2_ production compared with KC_Control_ (Fig. 4*B*, *bottom*). These findings suggest that the phenotype of isolated primary cells resembles what typically occurs *in vivo* under chronic alcohol intake (37, 39).

##### HMGB1 Induction, Translocation, and Secretion in Hepatocytes Is Critical for Alcohol-induced Liver Injury

Because of the high expression of HMGB1 *in vivo* in hepatocytes and to a much lesser extent in Kupffer cells, to validate this *in vitro*, we performed immunofluorescence analysis in H_Control_, H_EtOH_, KC_Control_, and KC_EtOH_. There was significant nuclear localization of HMGB1 in H_Control_, whereas H_EtOH_ showed increased nuclear HMGB1 expression and a punctate pattern of cytoplasmic HMGB1 suggestive of HMGB1 accumulation in the cytoplasm perhaps for subsequent secretion (Fig. 4*C*, *top*). To confirm that indeed secretion occurred, we performed Western blot analysis to detect HMGB1 in the culture medium, which validated the ability of hepatocytes to secrete a large amount of HMGB1 under ethanol treatment (Fig. 4*C*, *bottom*). KC_Control_ did not secrete HMGB1; however, induction of HMGB1 expression and secretion did occur but to a much lesser extent in KC_EtOH_ than in H_EtOH_ (Fig. 4*D*). Secretion was undetectable in stellate cells and sinusoidal endothelial cells despite nuclear HMGB1 expression (not shown). Moreover, H_Control_ were also responsive to ethanol treatment, secreting HMGB1 in a dose- and time-dependent fashion (Fig. 4*E*). Finally, because ethanol metabolism causes significant generation of reactive oxygen species in the liver, we asked whether hepatocytes will also increase HMGB1 secretion in response to prooxidant treatment. Both H_2_O_2_ and arachidonic acid increased HMGB1 expression and secretion in the absence of cell toxicity as validated by the 3-(4,5-dimethylthiazol-2-yl)-2,5-diphenyltetrazolium bromide assay (not shown); however, HMGB1 induction and secretion were prevented by co-treatment with the antioxidants catalase (to decompose H_2_O_2_) and vitamin E (to block lipid peroxidation reactions), respectively (Fig. 4*F*). Hence, HMGB1 induction and secretion in hepatocytes are oxidant stress-sensitive.

**FIGURE 5.**
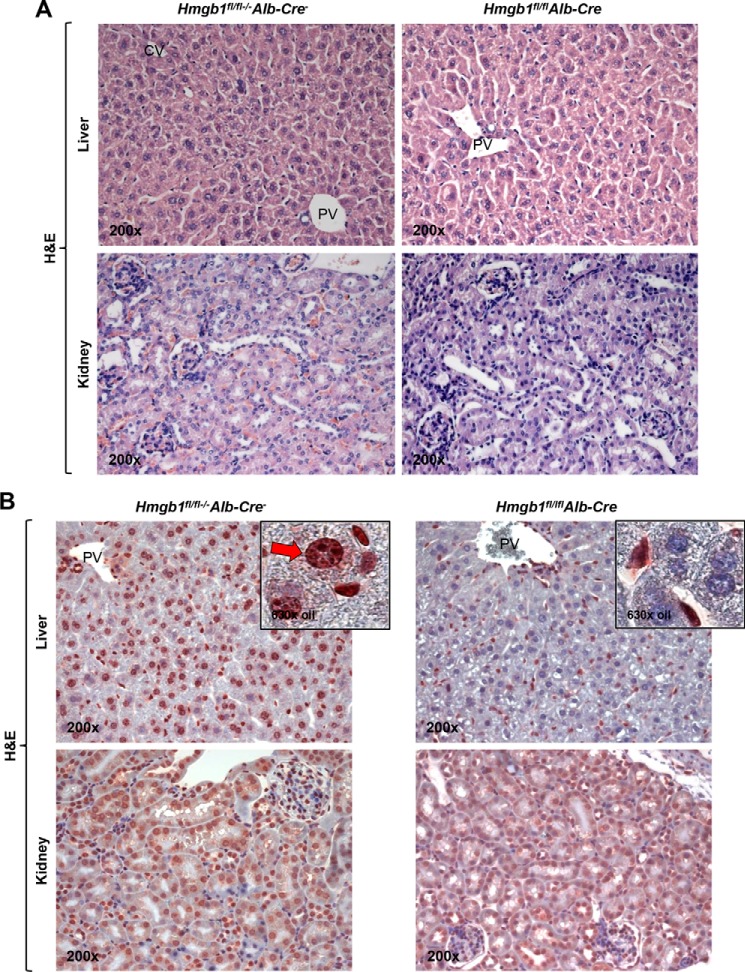

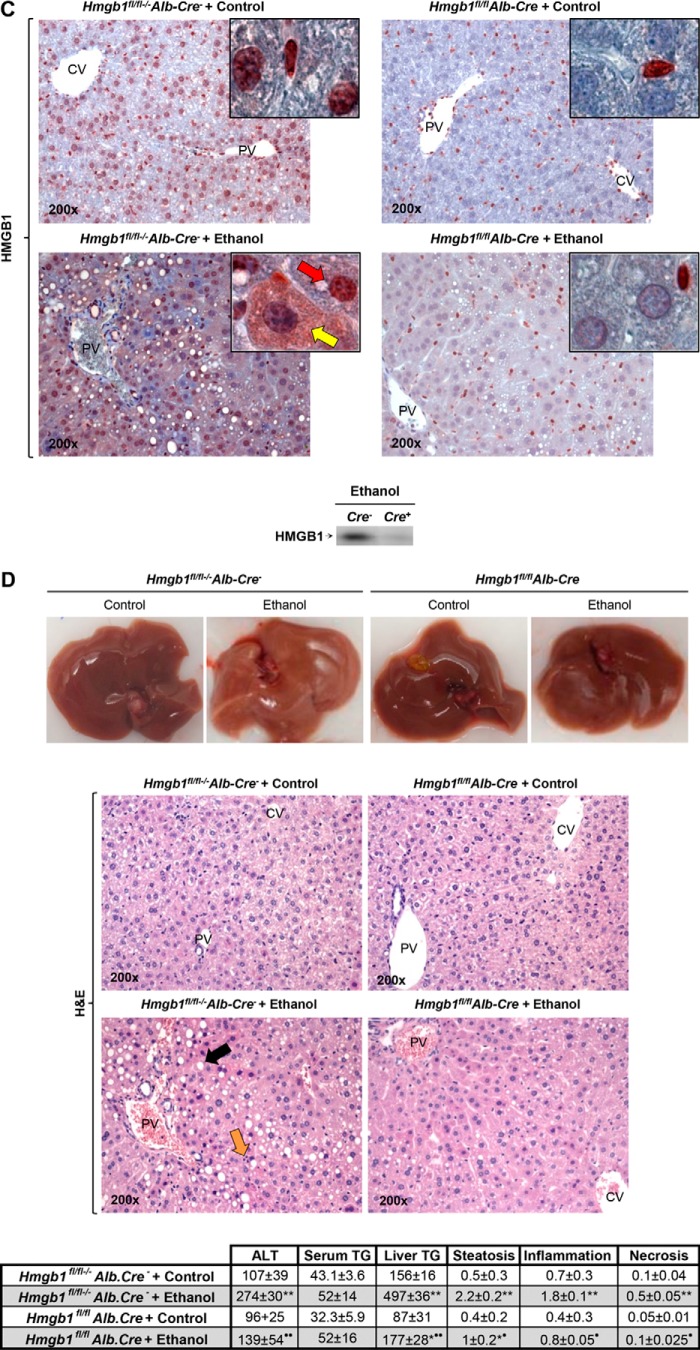

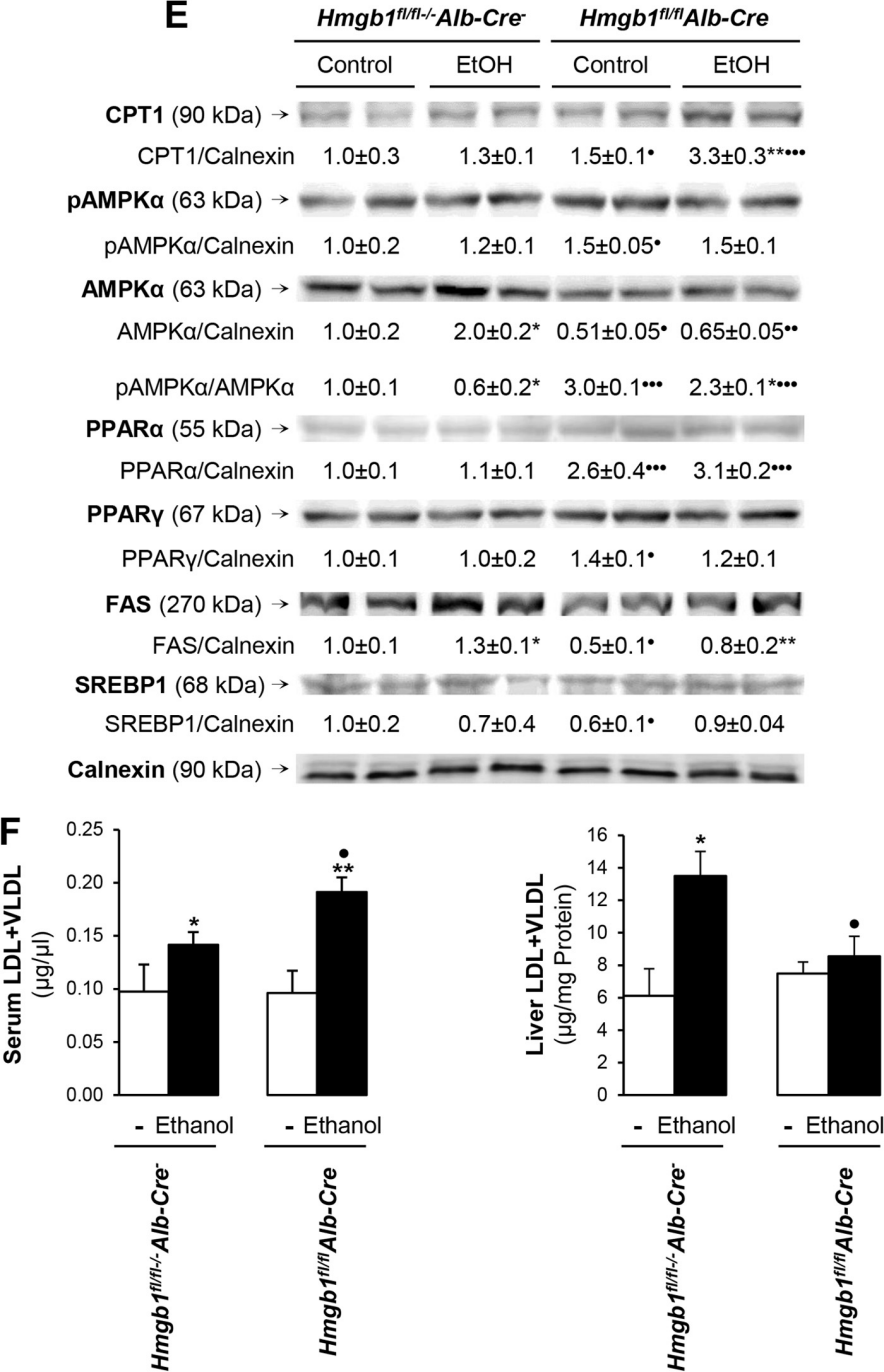
***Hmgb1^fl/fl^Alb-Cre* mice are protected from ALD.** H&E staining from liver and kidney from non-treated *Hmgb1^fl^*^/^*^fl^Alb-Cre* and *Hmgb1^fl^*^/^*^fl^*^−/−^*Alb-Cre*^−^ littermates is shown (*A*). HMGB1 IHC from liver and kidney from non-treated *Hmgb1^fl^*^/^*^fl^Alb-Cre* mice and *Hmgb1^fl^*^/^*^fl^*^−/−^*Alb-Cre*^−^ mice is shown (*B*). The livers from *Hmgb1^fl^*^/^*^fl^Alb-Cre* mice confirmed the absence of HMGB1-positive staining in hepatocytes compared with *Hmgb1^fl^*^/^*^fl^*^−/−^*Alb-Cre*^−^ mice (*red arrow*). *Hmgb1^fl^*^/^*^fl^Alb-Cre* and *Hmgb1^fl^*^/^*^fl^*^−/−^*Alb-Cre*^−^ littermates were fed for 7 weeks the control or the ethanol Lieber-DeCarli diet. In IHC for HMGB1, the *red arrow* indicates HMGB1 positive staining in the nucleus and negative staining in the cytoplasm, and the *yellow arrow* points to HMGB1 negative staining in the nucleus and positive staining in the cytoplasm (*C*, *top*). Western blot analysis for HMGB1 in the ethanol-fed mice only is shown (*C*, *bottom*). Images show the gross appearance of the livers from control and ethanol-fed *Hmgb1^fl^*^/^*^fl^Alb-Cre* and *Hmgb1^fl^*^/^*^fl^*^−/−^*Alb-Cre*^−^ mice (*D*, *top*). H&E staining shows steatosis (*black arrow*) and inflammation (*orange arrow*) (*D*, *middle*). Serum alanine aminotransferase (*ALT*) activity (units/liter), serum triglycerides (*TG*) (mg/dl), and liver triglycerides (mg/g of protein) along with the scores for steatosis, inflammation, and necrosis classified according to the Brunt scoring system (40) are shown (*D*, *bottom*). *n* = 10/group. *, *p* < 0.05; **p < 0.01 for ethanol *versus* control. •, *p* < 0.05; ••, *p* < 0.01 for *Hmgb1^fl^*^/^*^fl^Alb-Cre versus Hmgb1^fl^*^/^*^fl^*^−/−^*Alb-Cre*^−^. *Hmgb1* ablation increases CPT1, pAMPKα, and pPPARα expression and enhances LDL plus VLDL export, preventing steatosis and liver injury in mice. Western blot analysis revealed an increase in the expression of CPT1, pAMPKα, and pPPARα in ethanol-fed *Hmgb1^fl^*^/^*^fl^Alb-Cre* mice compared with *Hmgb1^fl^*^/^*^fl^*^−/−^*Alb-Cre*^−^ littermates (*E*). Serum and liver LDL plus VLDL levels in control and ethanol-fed *Hmgb1^fl^*^/^*^fl^Alb-Cre* and *Hmgb1^fl^*^/^*^fl^*^−/−^*Alb-Cre*^−^ mice are shown (*F*). Results are expressed as average values ± S.E. *n* = 10/group. *, *p* < 0.05; **, *p* < 0.01 for ethanol *versus* control. •, *p* < 0.05; •••, *p* < 0.001 for *Hmgb1^fl^*^/^*^fl^Alb-Cre versus Hmgb1^fl^*^/^*^fl^*^−/−^*Alb-Cre*^−^. *PV*, portal vein; *CV*, central vein.

Next, to determine whether HMGB1 induction and translocation in hepatocytes played a major role in the development of ALD, *Hmgb1^fl^*^/^*^fl^Alb-Cre* mice and control *Hmgb1^fl^*^/^*^fl^*^−/−^*Alb-Cre*^−^ littermates were used. In the absence of any treatment, these mice show normal liver and kidney architecture and no obvious phenotype as seen on H&E staining (Fig. 5*A*). Cell- and organ-specific *Hmgb1* ablation was validated by IHC, which confirmed the absence of HMGB1 mostly in hepatocytes and not in other cells or organs such as the kidney (Fig. 5*B*) (41). Next, *Hmgb1^fl^*^/^*^fl^Alb-Cre* and control *Hmgb1^fl^*^/^*^fl^*^−/−^*Alb-Cre*^−^ littermates were fed the control or the ethanol Lieber-DeCarli diet as described above. Ethanol-fed *Hmgb1^fl^*^/^*^fl^*^−/−^*Alb-Cre*^−^ mice showed enhanced HMGB1 expression and translocation (Fig. 5*C*) and developed significant liver injury and steatosis as shown macroscopically and by H&E staining, serum alanine aminotransferase, serum and liver triglycerides, and activity scores (steatosis, inflammation, and necrosis), whereas *Hmgb1^fl^*^/^*^fl^Alb-Cre* mice were protected from ALD (Fig. 5*D*). In summary, these data suggest that HMGB1 induction, translocation, and secretion in hepatocytes are critical for ALD.

##### Hmgb1 Ablation Increases Carnitine Palmitoyltransferase-1 (CPT1), Phosphorylated 5′-AMP-activated Protein Kinase-α (pAMPKα), and Phosphorylated Peroxisome Proliferator-activated Receptor-α (pPPARα) Expression and Enhances LDL plus VLDL Export, Preventing Steatosis and Liver Injury in Mice

To identify the mechanism whereby *Hmgb1* ablation protected mice from ALD, we analyzed the expression of the key proteins involved in fatty acid synthesis and fatty acid β-oxidation. Western blot analysis revealed an increase in the expression of CPT1, pAMPKα, and pPPARα, which are all involved in fatty acid β-oxidation, along with a slight decrease in fatty-acid synthase, which participates in fatty acid synthesis, in ethanol-fed *Hmgb1^fl^*^/^*^fl^Alb-Cre* mice compared with *Hmgb1^fl^*^/^*^fl^*^−/−^*Alb-Cre*^−^ littermates. However, the expression of PPARγ and SREBP1 remained unchanged (Fig. 5*E*). Moreover, there was enhanced LDL plus VLDL export from the liver to the serum in ethanol-fed *Hmgb1^fl^*^/^*^fl^Alb-Cre* compared with *Hmgb1^fl^*^/^*^fl^*^−/−^*Alb-Cre*^−^ littermates (Fig. 5*F*). Together, these results suggest that the up-regulation of CPT1, pAMPKα, and pPPARα along with the enhanced LDL plus VLDL export protects ethanol-fed *Hmgb1^fl^*^/^*^fl^Alb-Cre* mice, thus preventing hepatic steatosis and liver injury compared with their *Hmgb1^fl^*^/^*^fl^*^−/−^*Alb-Cre*^−^ littermates.

##### Identification of HMGB1 Isoforms in Mice and in Patients with ALD

Recent reports have highlighted the importance of PTMs within HMGB1 that can condition the mechanism of cellular HMGB1 secretion and/or its proinflammatory role (1, 30–35). Identification of these PTMs could contribute to understanding the pathogenesis of ALD and to developing new therapies. Therefore, we utilized proteomic MS to identify the HMGB1 isoforms in the liver and serum from mice fed the Lieber-DeCarli diets and the HMGB1 isoforms circulating in blood from alcoholic patients.

**FIGURE 6.**
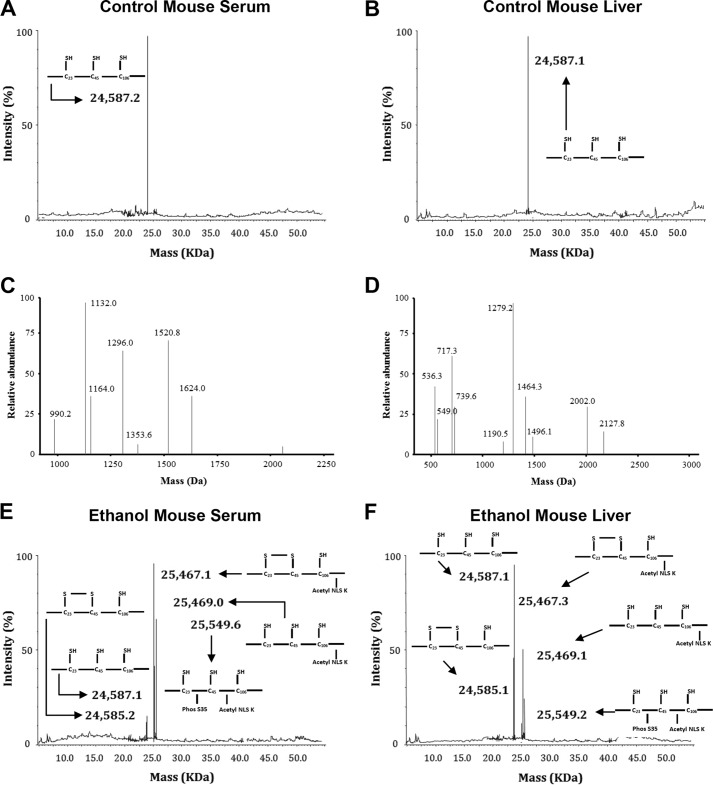

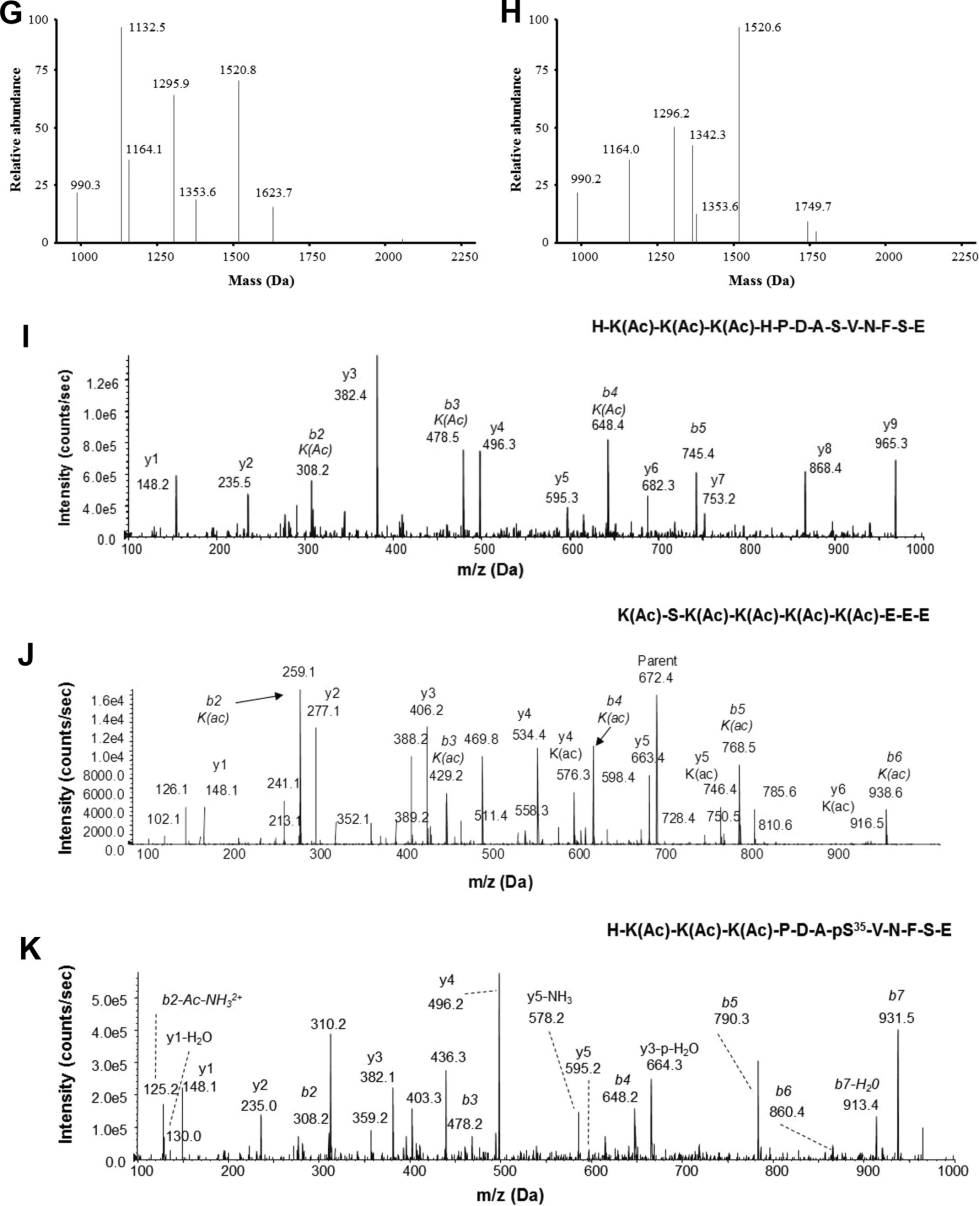
**Identification of HMGB1 PTMs in serum and liver from control and ethanol-fed mice.** Representative spectra of whole protein ESI-MS of HMGB1 isoforms isolated from either serum (*A*) or liver (*B*) from control mice are shown. Peptide MS of HMGB1 derived from control mouse serum or liver was performed following enzymatic digestion with either Glu-C to confirm acetyl status (*C*) or trypsin to confirm redox status (*D*). Representative spectra of whole protein ESI-MS of HMGB1 isoforms isolated from either serum (*E*) or liver (*F*) from ethanol-fed mice are shown. Peptide MS of HMGB1 derived from serum or liver from ethanol-fed mice was performed following enzymatic digestion with endopeptidase Glu-C to confirm the lack of acetyl modifications on the 24,587 and 24,585-Da isoforms (*G*) and the presence of acetyl modifications on the 25,467-, 25,469-, and 25,549-Da isoforms (*H*). Molecular weights and a schematic representation of each isoform are indicated on each spectra where required. Acetyl modifications were confirmed on lysine residues (shown as *K(Ac)*) after LC-MS/MS analysis for peptides spanning NLS1 (amino acids 27–39) (*I*) and NLS2 (amino acids 180–188 (179–187 minus methionine)) (*J*) in HMGB1 derived from mice fed ethanol. Phosphorylation modifications were confirmed on serine 35 (shown as *pS^35^*) after LC-MS/MS analysis of NLS1 (*K*) in HMGB1 derived from mice fed ethanol. The one-letter amino acid code is given for each peptide sequence, and *b* and *y* ions are highlighted with molecular weights where appropriate. Data are representative of at least six individual mice per group.

In control mice, we observed the presence of a single HMGB1 isoform with a molecular mass of 24,587 Da in both serum (Fig. 6*A*) and liver (Fig. 6*B*). MS analysis confirmed that the lysine residues within the peptides containing NLS1 (1624 Da) and NLS2 (1132 Da) were not acetylated (Fig. 6*C*). Furthermore, all cysteine residues were in the reduced state as shown by peptides of 1496 (cysteine 23), 549 (cysteine 45), and 2002 Da (cysteine 106) (Fig. 6*D*). This all-thiol isoform has been recently termed “fully reduced HMGB1” (48). These data are consistent with previously published observations from control mice (28, 46).

In serum and liver from ethanol-fed mice, the above described all-thiol, non-acetylated HMGB1 isoform was observed (Fig. 6, *E* and *F*). In addition, isoforms of 24,585, 25,467, 25,469, and 25,549 Da were detected (Fig. 6, *E* and *F*). MS analysis revealed that the 24,585-Da isoform was not acetylated in NLS1 and in NLS2 (presence of 1624- and 1132-Da peptides and absence of 1750- and 1342-Da peptides) (Fig. 6*G*). Contrary to this, MS analysis revealed that the 25,467-, 25,469-, and 25,549-Da isoforms were acetylated in NLS1 and in NLS2 (presence of 1750- and 1342-Da peptides and absence of 1624- and 1132-Da peptides) (Fig. 6*H*). Our previous reports have highlighted these peptides on representative spectra for unrelated findings (45, 50). Analysis of *b* and *y* ions generated following MS/MS analysis confirmed the acetylation of each lysine residue within NLS1 (Fig. 6*I*) and NLS2 (Fig. 6*J*). *b* and *y* ions were assigned to the corresponding amino acids according to our previously published methodologies (43, 44, 46, 51). The increased mass shift of 126 Da for NLS1 and of 210 Da for NLS2 represents the presence of either three or five acetyl modifications of either NLS1 or NLS2, respectively (Fig. 6, *I* and *J*). Additionally, MS/MS analysis of the 25,549-Da isoform revealed the presence of a phosphate group on serine 35 (Fig. 6*K*).

**FIGURE 7.**
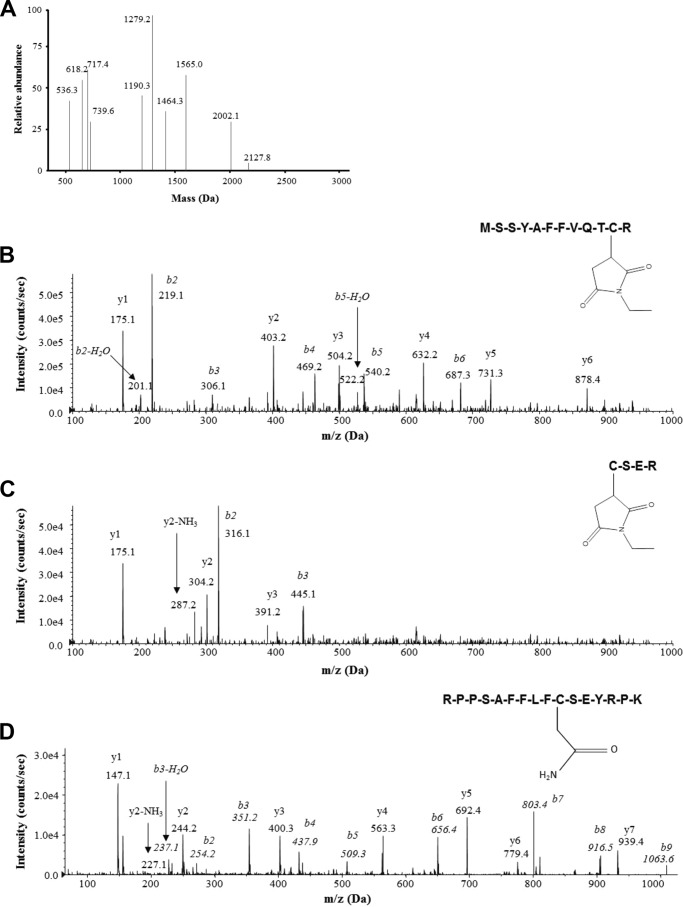
**LC-MS and LC-MS/MS redox characterization of HMGB1 isoforms in ethanol-fed mice.** Representative spectra of the LC-MS characterization of peptides produced from HMGB1 from the serum of ethanol-fed mice enzymatically cleaved with trypsin (*A*) are shown. Representative spectra of the LC-MS/MS of peptides derived from the tryptic digestion of HMGB1 isoforms derived from the serum of ethanol-fed mice covering cysteine residues 23 (*B*), 45 (*C*), and 106 (*D*), which had been subjected to alkylation by either *N*-ethylmaleimide or iodoacetamide, are also shown. The one-letter amino acid code is given for each peptide sequence, and *b* and *y* ions are labeled with molecular weights and amino acid code where appropriate. Data are representative of at least six individual ethanol-fed mice.

Regarding the redox characterization, we found that the cysteine residues within the 24,587-, 25,469-, and the 25,549-Da isoforms were reduced (as represented in Fig. 6*D*). In contrast, the 24,585- and 25,467-Da isoforms from the serum and livers of mice fed ethanol contained only peptides with masses of 1565 (cysteine 23) and 618 Da (cysteine 45), indicating the presence of an isoform recently termed “disulfide HMGB1” containing a disulfide bond between cysteines 23 and 45 (48), whereas cysteine 106 remained in the thiol state (Fig. 7*A*). In support of these data, MS/MS analysis of *b* and *y* ions generated from these peptides confirmed the amino acid sequence of each peptide and the addition of an *N*-ethylmaleimide (mass shift of 125 Da) adduct on cysteine 23 (Fig. 7*B*) and cysteine 45 (Fig. 7*C*) and an iodoacetamide adduct (mass shift of 57 Da) localized to cysteine 106 (Fig. 7*D*). In summary, for the analysis of HMGB1 from *in vivo* studies, schematic overviews of the PTMs identified on particular isoforms of HMGB1 are shown in Fig. 6, *A*, *B*, *E*, and *F* next to the corresponding molecular weight for ease of interpretation.

**FIGURE 8.**
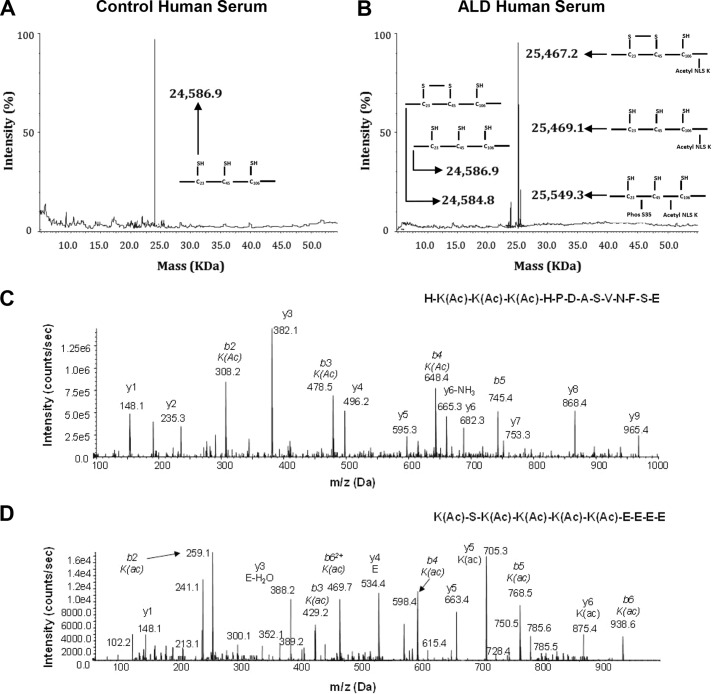
**Whole protein ESI-MS and LC-MS/MS characterization of HMGB1 isoforms in healthy volunteers and in patients with ALD.** Representative spectra of whole protein ESI-MS of HMGB1 isoforms isolated from serum of healthy volunteers (*A*) or patients with ALD (*B*) are shown. Molecular weights and a schematic representation of each isoform are indicated on each spectra where required. Representative spectra of the tandem MS characterization of a peptide containing lysine residues (Lys-28, -29, and -30) within NLS1 (*C*) and lysine residues (Lys-180, -182, -183, -184, and -185) within NLS2 (*D*) after Glu-C digestion of the 25,467- and 25,549-Da human HMGB1 isoforms following extraction from the serum of ALD patients are shown. HMGB1 was enzymatically cleaved with endopeptidase Glu-C to confirm the presence or absence of acetyl modifications on specific lysine residues (shown as *K(Ac)*) as described under “Experimental Procedures.” The one-letter amino acid code is given for each peptide sequence, and *b* and *y* ions are labeled with molecular weights and amino acid code where appropriate. Data are representative of either 10 healthy volunteers or 10 ALD patients.

As with the analysis of serum and livers of control and ethanol-fed mice, the same fingerprint of HMGB1 isoforms was observed in the serum of healthy volunteers as in the control mice (Fig. 8*A*) and in patients with ALD as in the ethanol-fed mice (Fig. 8*B*). The same PTMs on these isoforms were also confirmed by analysis of *b* and *y* ions generated during LC-MS/MS and are all summarized in Table 1. Representative MS/MS analysis showing lysine acetyl modifications for NLS1 and NLS2 are shown in Fig. 8, *C* and *D*. The actual presence of *b* and *y* ions generated in all LC-MS/MS experiments was compared with the predicted observations for that particular peptide sequence using the ExPASy bioinformatics portal. In summary, chronic alcohol intake induces PTMs in HMGB1; however, the main cellular source of acetylated, oxidized, and phosphorylated HMGB1 in the liver remained unknown.

**TABLE 1:**
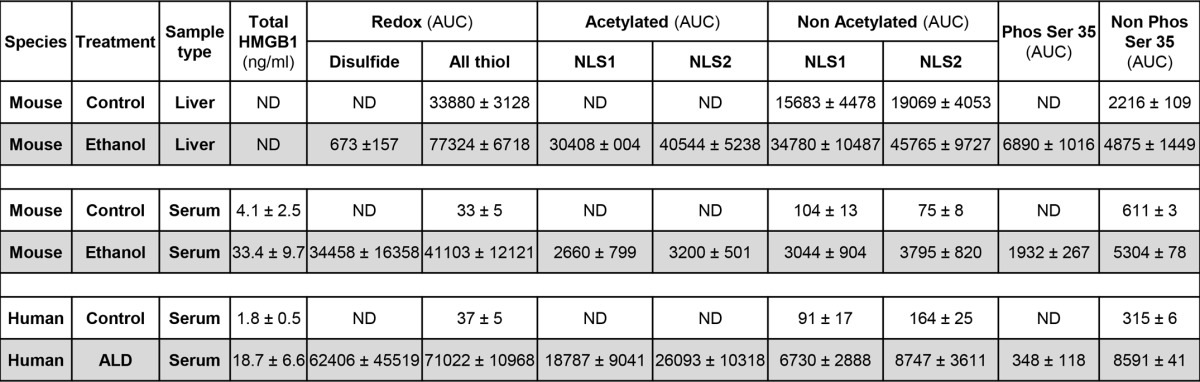
**Summary of the PTMs identified in HMGB1 in serum and liver from control and ethanol-fed mice and serum of healthy volunteers and alcoholic patients** PTMs were characterized by ESI-LC-MS as described under “Experimental Procedures.” With respect to redox characterization, “Disulfide” represents HMGB1 with a disulfide bond between cysteines 23 and 45 and a reduced cysteine 106, and “All thiol” represents HMGB1 with all cysteine residues reduced. “Acetylated” and “Non Acetylated” refer to the identification of lysines within NLS1 (lysines 28–30) and NLS2 (lysines 180 and 182–185) with and without acetyl modifications, respectively. “Phos Ser 35” and “Non Phos Ser 35” refer to the serine 35 with and without a phosphorylation modification, respectively. Relative quantification of HMGB1 isoforms is based on the area under the ESI-LC-MS curve (AUC) for a particular diagnostic peptide containing the key amino acid of interest for the PTM investigated. Extracted ion counts were normalized for total ion count. Peptides containing amino acids 13–24, 45–48, and 97–112 were used to quantify redox-dependent modifications, and peptides containing amino acids 26–39 and 180–188 were used for phospho and acetyl modifications, respectively. When no modification could be found, the lowest detectable area under the curve was recorded.

##### Hepatocytes Are the Major Source of Post-translationally Modified HMGB1

Because there was significant induction of HMGB1 under chronic alcohol consumption in hepatocytes and to a lesser extent in Kupffer cells, next, we asked which cell type could be the main source of post-translationally modified HMGB1 in the liver. Using the same strategy as that for liver and serum samples, MS analysis revealed that H_EtOH_ were the major source of total, oxidized, acetylated, and phosphorylated HMGB1, whereas KC_EtOH_ only produced acetylated and phosphorylated HMGB1 (Table 2). Thus, H_EtOH_ are not only the major source of native and post-translationally modified HMGB1, but they also recapitulate the three PTMs identified *in vivo* under ethanol consumption, whereas KC_EtOH_ only acetylate and phosphorylate HMGB1 but to a much lesser extent than H_EtOH_.

**TABLE 2:**
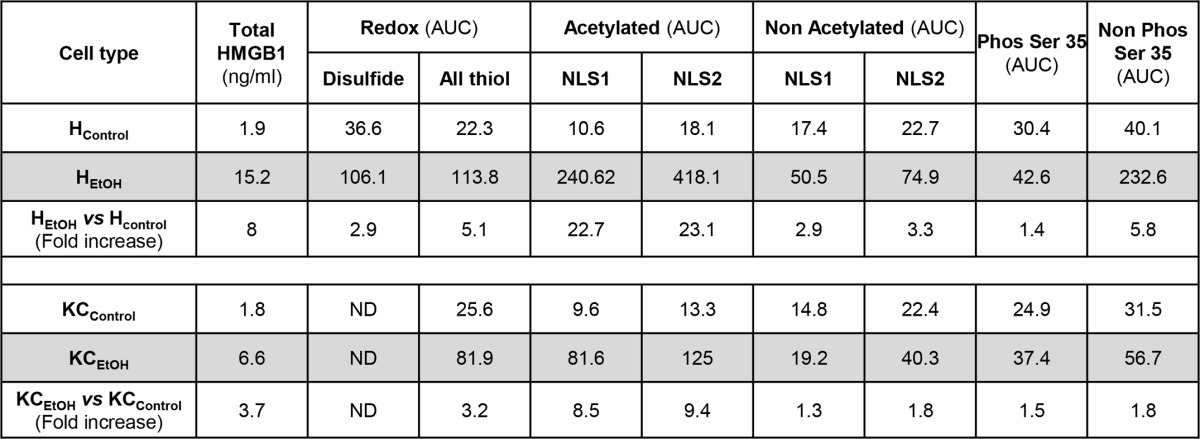
**Summary of PTMs identified in HMGB1 in hepatocytes and Kupffer cells isolated from control and ethanol-fed mice** PTMs were characterized by ESI-LC-MS as described under “Experimental Procedures.” With respect to redox characterization, “Disulfide” represents HMGB1 with a disulfide bond between cysteines 23 and 45 and a reduced cysteine 106, and “All thiol” represents HMGB1 with all cysteine residues reduced. “Acetylated” and “Non Acetylated” refer to the identification of lysines within NLS1 (lysines 28–30) and NLS2 (lysines 180 and 182–185) with and without acetyl modifications, respectively. “Phos Ser 35” and “Non Phos Ser 35” refer to serine 35 with and without a phosphorylation modification, respectively. HMGB1 isoforms were quantified based on the area under the ESI-LC-MS curve (AUC) for a particular diagnostic peptide containing the key amino acid of interest for the PTM investigated. Extracted ion counts were normalized for total ion count. Peptides containing amino acids 13–24, 45–48, and 97–112 were used to quantify redox-dependent modifications, and peptides containing amino acids 26–39 and 180–188 were used for phospho and acetyl modifications, respectively. The -fold increase in the area under the curve induced by ethanol treatment compared with control is also included for comparison.

## DISCUSSION

In the present study, we demonstrated for the first time that HMGB1 increases both in liver and serum from patients with acute ASH superimposed on ALD and cirrhosis and that there is correlation among the progressive increase in HMGB1 expression, translocation, and secretion with ALD stage in humans. In addition, in three mouse models of alcohol-induced liver injury, alcohol intake enhanced hepatic and serum HMGB1 expression, translocation from the nucleus to the cytoplasm, and secretion in mice similarly to what occurs in humans.

Using primary cell culture, we validated the ability of mouse hepatocytes to secrete a large amount of HMGB1 under ethanol treatment *in vivo* and in the absence of significant cell death. This also occurred, but to a much lesser extent, in Kupffer cells and was undetectable in stellate cells and sinusoidal endothelial cells. Furthermore, hepatocytes were also responsive to ethanol treatment *in vitro*, increasing, translocating, and secreting HMGB1 in a dose- and time-dependent fashion. HMGB1 secretion was oxidant stress-sensitive because prooxidants up-regulated HMGB1 secretion, and the increase was blunted by co-incubation with the antioxidants catalase and vitamin E.

We next demonstrated that HMGB1 induction and translocation in hepatocytes play a key role in the development of ALD because selective *Hmgb1* ablation in hepatocytes protected mice from alcohol-induced liver injury. This was due to increased expression of CPT1, pAMPKα, and pPPARα, key enzymes involved in fatty acid β-oxidation, along with efficient LDL plus VLDL export from the liver into the circulation, hence preventing steatosis and liver injury.

Moreover, we identified several PTMs from HMGB1 not previously described within the context of ALD whose functional consequences still remain unknown. We also showed that native and post-translationally modified HMGB1 are detected in serum from alcoholic patients and from ethanol-fed mice. Furthermore, under ethanol consumption, hepatocytes appeared to be the major source of native and post-translationally modified HMGB1, specifically of oxidized, acetylated, and phosphorylated HMGB1, whereas Kupffer cells only acetylated and phosphorylated HMGB1 although to a much lesser extent than hepatocytes.

Patients and mice with ALD showed the same pattern of acetylation, phosphorylation, and redox-dependent modifications in serum HMGB1. Furthermore, mouse liver samples showed acetylation and phosphorylation of HMGB1 in NLS1 in addition to acetylation of HMGB1 in NLS2. Moreover, evidence of disulfide bond formation between cysteines 23 and 45 was also observed, most likely as a result of the increased oxidative stress within the livers of these mice under alcohol consumption due to activation of cytochrome P450s and NADPH oxidase. A unique phosphorylation site in serine 35 localized in NLS1 was identified in all samples yet, its pathophysiological role warrants further investigation.

To date, the publication of the identified HMGB1 isoforms in samples from clinically relevant human inflammatory diseases is rather limited. Thus, these findings represent unique novel data as we describe for the first time the signature of HMGB1 isoforms present in serum during clinical ALD and in both serum and liver in a mouse model of ALD.

A key observation was the significant cytoplasmic HMGB1 staining in human and mouse samples from ALD. Although HMGB1 contains 43 lysines, only eight lysines in two key clusters appear to play a major role in preventing HMGB1 nuclear reentry due to PTMs: lysines 28–30 and lysines 43–44 representing a classic bipartite and functional NLS1 (1, 52) and lysines 180 and 182–185 within NLS2 (1, 52). Cells exclude HMGB1 from the nucleus by acetylating lysines, thereby neutralizing their basic charge and rendering them unable to function as NLSs. Thus, whether the identified ethanol-induced acetylation tilts the balance by inhibiting nuclear import, promoting HMGB1 accumulation in the cytoplasm perhaps for subsequent secretion, remains an open question (3).

Passive and active HMGB1 export to the extracellular milieu differs in that HMGB1 is highly acetylated for secretion, whereas passively released HMGB1 is not. Therefore, the identification of acetylated HMGB1 in the serum from alcoholic patients and in a murine model of ALD indicates that an active release of HMGB1 from hepatocytes and to a lesser extent from Kupffer cells, as shown in the culture medium from both cell types, may occur to drive ongoing inflammatory processes while perhaps serving as a translational biomarker of ALD; however, additional work is necessary to prove this. This PTM could reveal novel pathways for therapeutic intervention by targeting the acetylation process itself to block harmful secretion of HMGB1 during ALD.

In this study, we also detected significant oxidation of HMGB1. Recent evidence from preclinical and *in vitro* studies has revealed that the proinflammatory activity of HMGB1 is highly modulated by redox-dependent PTMs of three conserved cysteine residues at positions 23, 45, and 106. Cysteines 23 and 45 undergo conformational changes in response to oxidative stress and can form an intramolecular disulfide bond, whereas cysteine 106 is unpaired and has been shown to be essential for HMGB1 translocation from the nucleus to the cytoplasm (53) and for binding toll-like receptor-4 when present in its reduced state.

Similar findings were observed in ALD. Alcohol-induced liver injury causes significant oxidant stress due to alcohol metabolism via cytochrome P450 2E1; therefore, some HMGB1 oxidation was anticipated to occur in this setting. Besides, a more oxidizing environment due to the increase in reactive oxygen species and to GSH depletion under alcohol intake would favor HMGB1 oxidation.

Recruitment of neutrophils and macrophages and their activation to release proinflammatory cytokines contribute to sterile inflammation in ALD. It is possible that HMGB1 may play a role in this process by switching among mutually exclusive redox states. With all cysteines reduced, HMGB1 could act as a chemoattractant, whereas a disulfide bond between cysteines 23 and 45 could enable toll-like receptor-4 interactions and eventually promote cytokine production. Terminal oxidation to sulfonates by reactive oxygen species could then abrogate both activities (51). In ALD, HMGB1 could likely be completely reduced at first and disulfide-bonded later due to the alcohol-induced oxidative burst. Thus, HMGB1 could orchestrate both events (leukocyte recruitment and their activation to secrete inflammatory cytokines by adopting mutually exclusive forms) in sterile inflammation in ALD. However, additional studies are necessary to validate this speculation. Interestingly, we noted that *in vitro* only hepatocytes, but not Kupffer cells, released disulfide-bonded HMGB1 in response to ethanol, suggesting that they could be the initial source of mediators that could drive cytokine production in ALD.

Finally, within this current investigation, we also reported the novel identification of phosphorylated serine 35 in HMGB1 in both human and murine serum and liver samples from ALD. Phosphorylation in the two NLSs is key for the nuclear to cytoplasmic transport and secretion of HMGB1 (54). Of 14 potential phosphorylation sites, six are located within NLSs, but only serine 35 appeared phosphorylated in ALD. Ethanol-induced HMGB1 phosphorylation could increase the thermal stability of HMGB1, decrease its affinity for linear DNA (55), and favor binding to the nuclear exportin CRM1 for migration to the cytoplasm (1) in addition to disrupting the interaction of HMGB1 with nuclear importin (34). Phosphorylation of serines 35, 39, and 42 in NLS1 in HMGB1 is critical for HMGB1 translocation into the cytoplasm (56). HMGB1 serine residues can be phosphorylated by TNFα and is then relocated toward secretion and does not re-enter the nucleus (54). Because of the major role of TNFα in ALD, it is possible that it may be a driver of HMGB1 phosphorylation and eventually secretion. Future studies will clarify this hypothesis. Lastly, phosphorylation of serines 35, 53, and 181 by PKC-ζ has been described to enhance HMGB1 secretion in HCT116 colon cancer cells (57).

In summary, the data presented here implicate a potential key role of HMGB1 in the pathogenesis of ALD. Furthermore, the novel identification of mostly hepatocyte-derived HMGB1 isoforms may provide enhanced mechanistic insights to identify novel targets for therapeutic intervention or biomarkers to enable patient stratification and eventually treatment.

## Footnotes

4 The abbreviations used are:

ALD: alcoholic liver disease
ASH: alcoholic steatohepatitis
CPT1: carnitine palmitoyltransferase-1
ESI: electrospray ionization
H_Control_: hepatocytes from mice fed control diet
H_EtOH_: hepatocytes from mice fed ethanol diet
HMGB1: high mobility group box-1
IHC: immunohistochemistry
KC_Control_: Kupffer cells from mice fed control diet
KC_EtOH_: Kupffer cells from mice fed ethanol diet
MS/MS: tandem mass spectrometry
NLS: nuclear localization signal
pAMPKα: phosphorylated 5′-AMP-activated protein kinase-α
pPPARα: phosphorylated peroxisome proliferator-activated receptor-α
PTM: post-translational modification.
